# Perspective distortion tolerances and skull-face registration in craniofacial superimposition: an analytical review

**DOI:** 10.1007/s00414-023-03081-3

**Published:** 2023-09-13

**Authors:** Sean S. Healy, Carl N. Stephan

**Affiliations:** Laboratory for Human Craniofacial and Skeletal Identification (HuCS-ID Lab), School of Biomedical Sciences, The University of Queensland, Brisbane, 4072 Australia

**Keywords:** Forensic anthropology, Photographic superimposition, Video superimposition, Alignment, Error tolerance, Face

## Abstract

Craniofacial superimposition requires the photographic registration of a skull at transparency to a photograph of an antemortem (AM) face so that anatomical concordance between the two can be assessed. When the camera vantage point of the AM photograph is exactly replicated for skull photography, the superimposition is a relatively straightforward process as the images are precisely comparable without complicating factors. In practice, however, focus distances are almost never exactly replicated because the focus distance for AM face photography is rarely known. Embedded differences in perspective, thereby, drive the images away from correspondence, raising questions as to how much difference can be tolerated and what image registration methods should be used. Recently, a ± 1% mismatch in facial height has been posited as an acceptable upper tolerance limit to differential perspective, but this proposition is speculative and has not yet been confirmed by tests on real-life images. In addition, the impact of image registration methods, though critically relevant, has received comparatively little consideration. This paper provides the first in-depth review of these intertwined perspective/registration matters and objective evaluation of tolerances by using real 2D photographic images and synthetic images generated from 3D CT data to demonstrate perspective impact on skull morphology. Taken together, the review confirms a ≤ 1% perspective difference in facial height to be a suitable criterion for craniofacial superimposition (at least as a starting point for method improvement), and that image registration should be point-based using a sellion/nasion combination to minimize anatomical misalignment in the principal region-of-interest (the mid-face).

**Supplementary Information:**

The online version contains supplementary material available at 10.1007/s00414-023-03081-3.

## Introduction

Craniofacial superimposition is a forensic method that relies on the overlay of still-frame or motion-picture images of a skull and face, at partial image transparency, so the degree of anatomical correspondence between the two can be assessed [1–12]. This method has long been used in legal medicine, typically in the forensic anthropology/odontology setting, when other, more reliable methods such as genetic testing, medical record comparison, or fingerprint analysis are not possible [13–15]. Although superimposition has seen use in an identification context since its inception, in recent years (due to the method’s unreliability), it has been strongly recommended to purely be restricted to the exclusion context as a screening tool only [8, 16–19].

As superimposition methods are most frequently undertaken in the Western world after other more reliable methods have first been attempted, the need for the method to be scientifically sound is heightened as it cannot be used with other cross-checks or confirmations in a corroborative context [20–22]. Note here that recommendations to use multiple methods to strengthen unreliable superimposition results have been made [23]; however, this is not possible when superimposition methods are used as a “last resort” or a “last ditch effort” for skeletal identification.

The need for scientifically sound and dependable superimposition methods is saliently underscored by recently attempted (but failed) reverification of superimposition identification results by other higher-powered methods in special cases where these higher-powered methods became feasible later on. For example, genetic testing detected a 70% misidentification rate for the Patio 29 mass grave, concerning a total of 69 total superimpositions [24]. Erroneous casework exclusions recognized via subsequently conducted radiographic comparisons [25, 26] also raise concern for accurate results and show that the use of these methods for exclusion, or as a screening tool, is no safety net for unreliable techniques (true matches may be missed). This is illustrated by the bodies-in-barrels murders, where a false negative superimposition made early in this serial killing case was not ideal [25, 26].

While the overarching principles and aim of superimposition methods are widely agreed, how the methods are implemented in practice is highly varied [27, 28]. This lack of standardization partly results because scientifically valid method fundamentals have not yet been adequately elucidated, leaving room for multiple interpretations, speculations, and implementations. For example, in the craniofacial superimposition research domain, the majority of research attention is awarded to the end-stage anatomical comparison (see, e.g., [14, 29, 30]), with other precursor factors receiving little attention [20, 21]. While anatomical evaluations of the skull and the face are important and interesting, this task is the last of a long change of perquisite steps that must be completed correctly for a valid superimposition to be conducted. While efforts to increase standardization of highly varied present-day methods are laudable [28, 31], these alone are unlikely to successfully resolve superimposition’s accuracy and reliability problems without address of the other gaps in method foundations. This also applies to those methods that some practitioners proclaim are esteemed and/or scientifically valid in their fullness [32, 33]—since they too fail to address fundamental prerequisite steps [11, 20–22, 34–36].

An excellent case in point is the long avoidance of using the focus distance variable for face photography in craniofacial superimposition—because it has been hailed as far too difficult, if not impossible, to estimate [11] p. 122–3, [34] p.118, [35] p.240. Focus distance is critical because it, in part, sets the perspective of the face or skull in the photograph [6, 37–39] as one of the factors of camera vantage point, just like head position. If ground truth matching skull and face photographs are to correspond in a 1:1 anatomical format, they must be photographed from the same vantage point, so their perspective is identical, or at least very similar [37–39]. Departure from comparable focus distances for the skull and face photographs will compromise the superimposition result because the perspective difference changes the morphological appearance of the skull/face relative to one another and away from their ground truth correspondence (if the pair are, in fact, a match).

Recent advances have shown that focus distance estimation from photographs of faces without scales is not impossible [21, 36]. The palpebral fissure length has, for example, been recognized to be consistent enough between individuals of the same age and sex that a sample mean can be used in place of an individual’s real-world value to make an estimation when the focal length of the lens used to acquire the photograph is known [20–22, 40]. This development provides the first starting point for matching the perspective of a skull to a facial photograph, as necessary for undertaking 1:1 anatomical comparison in craniofacial superimposition.

While the focus distance is a key prerequisite to satisfy in craniofacial superimposition prior to anatomical comparisons, it is not the only variable deserving attention. For example, because the focus distance estimation for the skull will likely hold a degree of error relative to the antemortem face photograph [21], how the two images are registered becomes yet another important factor. The registration method may minimize or exaggerate the misalignment in the principal region-of-interest (the face) for the craniofacial analyses. Tolerances for perspective distortion mismatch (or error), and what registration methods optimize results, are critical to establish and understand. So far, they have, however, received little attention.

For the initial formulation to estimate the focus distance [21], a tolerance for focus distance error was set using a ± 1% difference in the mean linear face height measured from trichion [tr′] to menton [m′] (= 179 mm [41]). Since the formulation concerns a chord length after Titlbach [38], it should be noted that it discounts the perspective effect of not being able to see around the edges of curved structures at short focus distances, which also applies [39, 42, 43]. This is, in part, a limitation that makes it valuable to review the previously suggested ± 1% perspective mismatch tolerance level with real-world face/skull photographs.

The aim of this paper is to review how focus distance mismatches impact the morphological appearance of the head and image registrations, using real face photographs, to determine if the ± 1% difference in facial height (as previously recommended) is a good criterion. We further generate synthetic 2D images from a 3D CT scan of a living subject using OsiriX^®^ [44] and Blender^®^ [45] to demonstrate and review the effects of focus distance mismatches on a ground-truth skull/face pair, allowing the suitability of the ± 1% criterion to be further evaluated.

## The ± 1% tolerance for perspective mismatch in craniofacial superimposition

Before putting the tolerance for ± 1% perspective mismatch to the test, it is worth reviewing, for clarity, its primary basis of derivation and its positive and negative attributes.

### Derivation

**Fig. 1:**
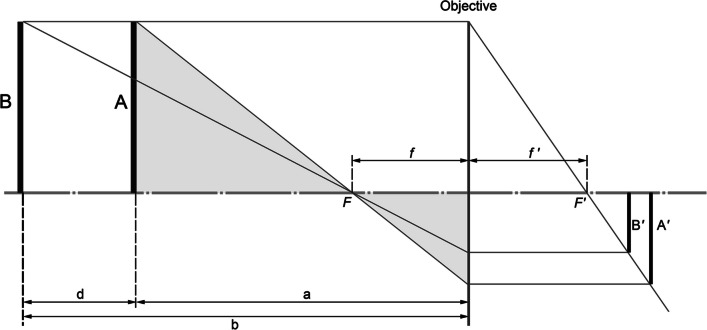
Titlbach’s [38] geometrical summary of perspective distortion during point projection of a 3D scene to a 2D film plane. A and B represent objects of identical real-life size, but they fall at different distances from the objective lens of the camera (a and a + d, respectively). F is the focal point and f is the focal distance. A′ and B′ are the film plane representations of A and B—note they are at different lengths. Image reproduced from [39] p.520.e5 with permission from Elsevier

The derivation rests entirely on the apparent change in size at the image receptor of two identical chord lengths placed at different focus distances from the camera (Fig. 1). This mathematical relationship, based on laws of similar triangles, was first described by Titlbach [38] and extended in [21]. The longest vertical chord length of the face (tr′– m′, per abbreviation conventions of [46]) is used as variable A/B (after Titlbach) to quantify the perspective mismatch between two images resulting from the use of different focus distances (i.e., the subject to camera distances).

### Positive attributes

Using a large chord length, such as face height, is favorable because measurement errors become relatively smaller in contrast to the overall measurement length. Being easier to measure than a shorter chord length, the facial height also provides a more sensitive measurement of the focus distance change and the perspective mismatch than smaller linear distance measurements.

The percent unit descriptor is useful because it universally applies to all focus distance comparisons, awarding the metric high utility. In contrast, the error difference of the focus distance in meter units cannot be used for the same purpose because it is not linearly proportional to the perspective mismatch [39]. That is, for a given meter difference in the focus distance, the perspective effect will be less at longer focus distances than shorter ones [39].

**Fig. 2:**
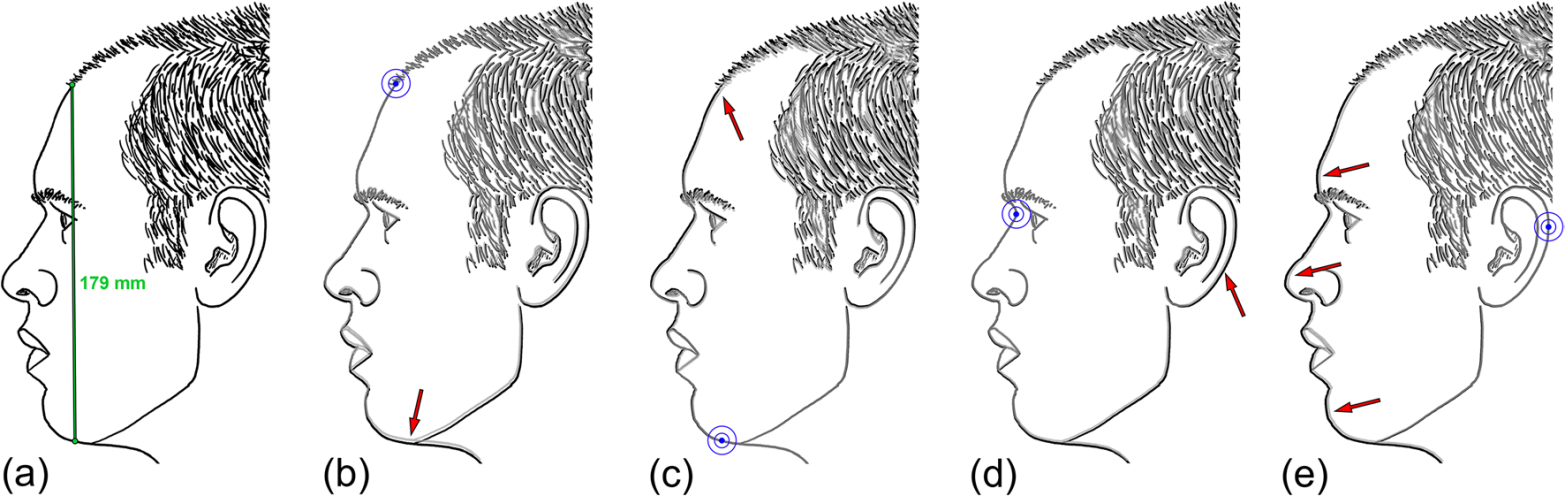
Anatomical misalignments of − 1% reduced (facial height: tr′–m′) outlines and at a variety of different point registrations for the superimpositions. Here, duplicate outlines of the face photograph at one focus distance are used so that differences can be saliently evaluated. **a** Raw image scaled to trichion-mention (tr′–m′) mean distance reported by Farkas [41] = 179 mm (1:4 image size reproduction here). **b** Raw outline (black) and 1% reduced duplicate (grey), registered on trichion [tr′] (blue target). NB. Misalignment increases throughout the face and is largest, where the full − 1% change is recorded at the opposite end of the chord length, e.g., at menton [m′] (red arrow). **c** Raw outline (black) and 1% reduced duplicate (grey), registered on menton [m′] (blue target). NB. Misalignment reverses compared to **b** and now increases throughout the vertical height of the face and is largest (∆1%) superiorly, e.g., at trichion [tr′] (red arrow). **d** Raw outline (black) and 1% reduced duplicate (grey), registered on sellion (se′) (blue target). NB. Misalignment is now shared across tr′ and m′ and reduced to half of 1% at each vertical extreme of the face. Misalignment is now greatest at ears (red arrow), rather than at the anterior facial profile. **e** Raw outline (black) and 1% reduced duplicate (grey), registered on postaurale (pa′) (blue target). NB. In contrast to **d**, the ear superimposes with less error, but now, the misalignment of the entire anterior face profile increases (red arrows)

Assuming central placement of the skull and face in the field-of-view of the camera for photography, registration of skulls/faces using the mid-face will effectively half the total linear metric error within the facial plane because the error size resulting from differential perspective of the chord length is spread from the center of the face outwards, rather than additively accumulating throughout the object length relative to one precisely registered end and being realized in complete fullness at the opposite end (Fig. 2). Subsequently, the ± 1% tolerance is halved in practice to a maximum of ± 0.5% in any facial region. For the facial height (tr′– m′), this translates into ± 0.9 mm maximum misalignment along the anterior vertical plane of the face (Fig. 2).

For craniofacial superimposition, since the skeletal face is slightly smaller than the head’s face height, using the face height provides a conservative estimate of the maximum amount of perspective difference present overall across the skull. That is, all else being equal, it will tend to provide slight overestimations of the errors resulting from differential perspective.

### Limitations

As mentioned above, the ± 1% perspective mismatch criterion is based on image receptor views of a chord. The resulting size difference does not take into account the inability to see the end points when they fall on curved surfaces and as viewed at short focus distances [39, 42]. Thus, the ± 1% perspective mismatch criterion represents a simplified assessment of a more complex perspective problem.

By using a mean face height universally for all individuals (to facilitate convenience), the perspective estimate will be an approximation for some individuals whose true face height value is far from the mean.

## Image registration factors compelled by perspective differences

When a different vantage point is used to acquire a photograph of a subject (even if the subject remains entirely stationary and only the distance between the subject and the camera varies), the two resultant images will never align exactly at superimposition because the view of the subject has inherently been altered [20–22, 37–39]. Not only is the scale of the subject within the field-of-view affected, but so are the relations of the subject’s facial features to one another [20–22, 37–39]. This complicates the registration process for superimposition because there is a lack of homology between focus distance mismatched images, even if they are derived from the same subject. In such cases, there are two options for the image registration to permit the superimposition process to continue: (1) minimize the differences between the head structures globally, so all parts of the head contain some error; or (2) minimize the error for features in the principle-region-of-interest (PRI) at the sacrifice of larger errors in other head regions.

For craniofacial superimposition, where the PRI is the anteriorly located face (not the back of the head, which is often covered with hair and prevents evaluation of skull/head contours), there is merit to selecting a mid-facial registration point to minimize the perspective mismatch in the mid-anterior face where most major facial features fall (Fig. 2). In contrast, if a registration point far from the face is selected, then the perspective differences will be manifested more intensely in the face region (Fig. 2). With respect to a facial registration point, it is worth noting that it should fall close to the middle of the PRI to again avoid exaggerating the effects of the perspective distortion mismatch as happens when a landmark at the extremes of the PRI (e.g., pronasale, trichion, menton, or zygion, respectively) is used.

When undertaking a superimposition, every effort should be made to replicate the focus distance used for AM face photography to acquire the skull photographs. Mid-face point registration should only be used in a secondary capacity to minimize any residual focus distance error effects. By minimizing the differences for the face alignment, this point registration opens the opportunity to use images with greater amounts of perspective error than could otherwise be permissible with a least-squares type of global registration.

**Fig. 3:**
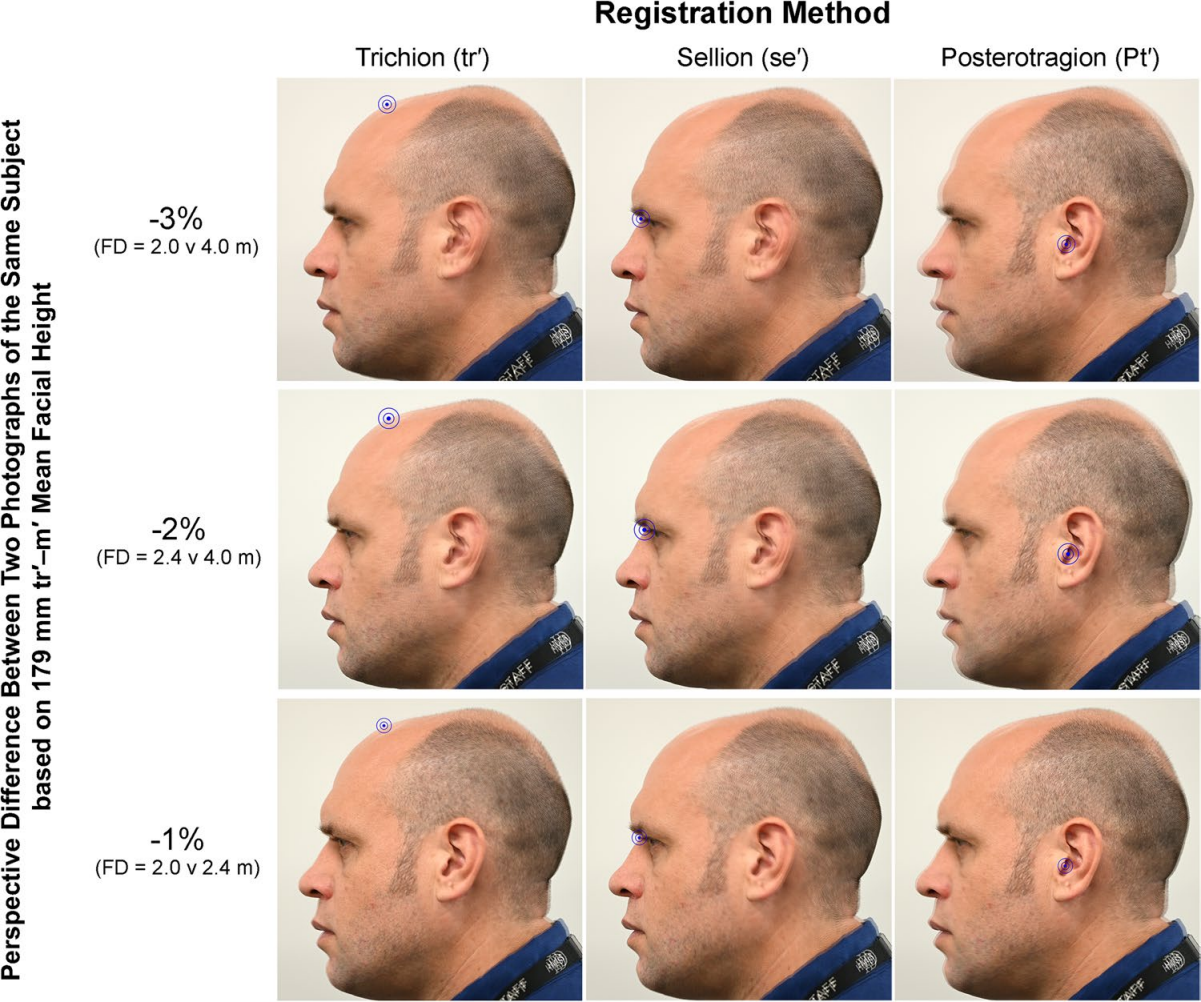
Anatomical misalignments of facial photographs of the same subject in two photographs taken at different focus distances and registered for superimposition using different facial landmarks. In each instance, the larger focus distance image is superimposed on the smaller focus distance image at an opacity of 50%. All images were acquired with a Nikon^®^ D780 camera body fitted with a Nikon^®^ AF-S 105 mm f/2.8 prime lens and on the same day (one after another without relocation or repositioning of the subject). FD = Image focus distance. Blue target highlights the registration point/landmark

To remain valid, the anatomical mismatch at regions far from the registration point should not be so great when using the center PRI registration point, as to prevent the evaluation of key, but more distant, face regions. An excellent illustration of this consideration is, for example, the position of the ear and external auditory meatus in profile view when two images of a face have been registered at sellion (Fig. 2). Perspective mismatch should be small enough that the sellion registration does not produce large misalignments at the ear that prevent its evaluation. These items are most clearly visualized using a single object (subject’s face) at different focus distances with repeat photographs. In these instances, the perspective mismatch manifests itself as a dual silhouette of the face with some offset—an effect that we refer to as *shadowing* (Figs. 2 and 3). This shadowing is unwanted in superimposition, and it applies as much to skull/face pairs as it does to face-to-face pairs. When conducting a superimposition, it is ideal if there is no perspective mismatch and thus no shadowing. In reality, this however will rarely be obtained (at least in craniofacial superimposition) since the focus distance used for skull photography must be estimated from the face photograph, such that shadowing effects of perspective mismatch are almost always present—if only to a small degree in ideal cases.

**Fig. 4:**
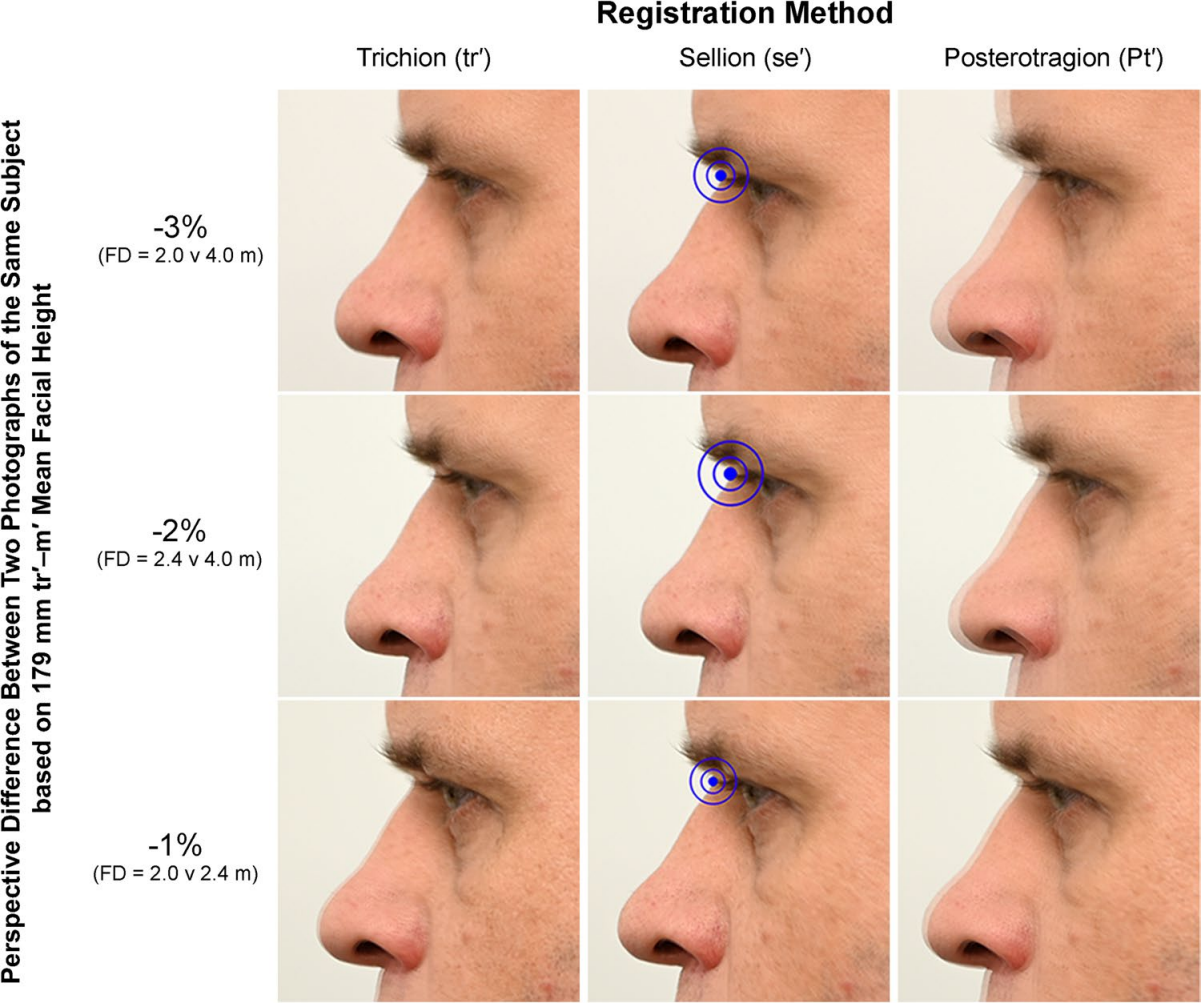
A zoomed view (310% enlargement) of the eye/nose region of each image panel in Fig. 3 to facilitate detailed evaluation of photographic misalignments at the mid-face resulting from perspective and registration differences. Note that the best alignment of the face for the two images providing near exact superimposition and without readily apparent shadowing of silhouettes is the 1% perspective difference condition registered at sellion (bottom row, middle image). All images were acquired with a Nikon^®^ D780 camera body fitted with a Nikon^®^ AF-S 105 mm f/2.8 prime lens and are taken on the same day (one after another without relocation or repositioning of the subject). FD = Image focus distance. See Fig. 2 for illustrations of the registration points used for each image superimposition

It is important to note that even proximally located features on a *2D image* may exhibit large magnitudes of perspective-induced change if, in *3D space*, the distance from the registration point is large. This, for example, applies to the eyes where, in 2D profile views, they appear proximal to sellion (a plausible registration point in the PRI), but in 3D reality, the exocanthion of the eyes is quite far laterally from this landmark due to distance along the axis parallel to the camera line-of-sight in 3D space. Subsequently, even with sellion registration to enhance alignment of the median outline of the face, the seemingly proximal eye region on the 2D image may not benefit so much from the sellion registration maneuver (Figs. 3 and 4).

Under no circumstances should two images in craniofacial superimposition be aligned using multiple different registration points in different local regions to produce better alignment of those regions as might be achieved in stepwise or serial fashion across the entire face. If perspective mismatches exist to the extent that multiple registration points are necessary, the skull/face pair should be designated as a non-matching set. Superimposition enhancement using a single point registration should only be employed when the degree of perspective mismatch is small (i.e., 0 to ± 1%). When using multiple face images taken at different camera vantage points, it is advisable to register the image suite using the same registration point across all images, so that they are consistently aligned in comparable manner. This requires the common registration landmark to be clearly visible across all images.

In this review, we reserve the word “registration” to describe only one component of the stepwise superimposition process where the test object (skull) is aligned, in its fullness, to the reference object (antemortem face image), thereby enabling anatomical comparison. That is, registration represents the first initial step that sets the test object’s position relative to the reference object. This excludes and precedes the other separate steps of determining the skull’s precise pitch, roll, and yaw. This is an important distinction. So far, skull alignment in the superimposition literature universally refers to pitch/roll/yaw adjustments (see, e.g., [6, 14, 23, 34]) without consideration for independent setting of the skull position using a precisely described registration procedure. Pitch/roll/yaw should be fine-tuned as a secondary step to this registration, ensuring the nasion/sellion registration is not lost in the rest of the skull alignment process.

While it is beyond the scope of this paper to review the validity of setting pitch/yaw/roll of the skull in superimposition, it is worth noting that methods are highly varied and a single field-wide agreed method has, so far, not been forthcoming [31]. There are however some commonalities of approach that are worth listing. For example, skulls and faces are often positioned in reference to.

1. Alignment of whitnall’s tubercles/exocanthions [14, 16, 17, 34];
2. Alignment of external auditory meatus (or porion) with tragus or soft tissue auditory canal [16, 17]; and
3. A plane joining subnasale and menton [14, 17].

## Is the ± 1% tolerance for perspective mismatch based on mean facial height sufficient and is a single point registration at sellion helpful?

To address these questions, we limit ourselves to the profile view. This is justified on four grounds: (1) this view has been regarded as being the most informative for craniofacial superimposition [29]; (2) this view is highly sensitive to single point registration effects as it presents the longest antero-posterior distances of the head; (3) this view is recommended to be used to supplement frontal or near frontal views wherever possible [29]; and (4) what works for profile views easily cross-apply to frontal views for median plane registration landmarks.

A series of different focus distance combinations were used to yield images of the same subject with 1, 2, and 3% differences per a 179 mm linear length following the similar triangle theory elucidated by Titlbach [38] and as recommended by Stephan [21, 39], to demonstrate the effects of perspective mismatch at skull superimposition. So that the impact of image registration landmarks could simultaneously be demonstrated in addition to the perspective mismatch, in each superimposition instance, the two face images were separately registered using three different landmarks (trichion, sellion, and posterotragion after definitions by [46]) for a total of nine superimpositions (Fig. 3).

At − 3% perspective difference, when the images are registered on posterotragion (which is close both to the head and image center), a large degree of double face outline (shadow) is clearly evident around the head. The silhouette of the face is misaligned by 4.0–4.5 mm down the majority of the anterior face profile, which is the equivalent of 82% of the mean tissue depth at g–g′, 60% of the depth at n–se′, and 38% of the tissue depth at pg–pg′ (per global mean facial soft tissue thickness values reported by [47]). By far, this represents too much error to permit accurate assessment of the skull and face contours in craniofacial superimposition (Fig. 4). Registration of the images at sellion fails to relieve much of the mismatch at the − 3% level (Fig. 4).

At − 2% perspective difference between the images and when registration is based on posterotragion, a shadow still exists around the head (Fig. 3) with misalignment at the face by 2.5–3.0 mm. This is somewhat smaller than the error at − 3% perspective difference, but still substantial, and registration on sellion exhibits clear mispositioning of the ear, meaning that porion (an important landmark for pitch/yaw/roll estimation per descriptions above) could not be reliably used in the superimposition procedure (Figs. 3 and 4).

At − 1% perspective difference and when registration is based on the posterotragion, there is very little shadow around the head margins and only a 1.0–1.5 mm misalignment down the median edge of the face (Figs. 3 and 4). This misalignment represents 27% of the mean tissue depth at g–g′, 20% of the depth at n–se′, and 13% of the tissue depth at pg–pg′, per global mean facial soft tissue thickness values reported by [47]. This represents a substantial improvement on the − 3% error context and with image registration at sellion, the alignment of the face becomes near-exact (Fig. 4). Ear structure/position is retained with crisp margins and little shadow compared to the − 2% and − 3% conditions. These results confirm the ± 1% difference in perspective distortion as a sufficient upper limit for superimposition casework and that a nasion/sellion registration indeed enhances the superimposition by minimizing the visual impact of the perspective difference in the PRI.

**Fig. 5:**
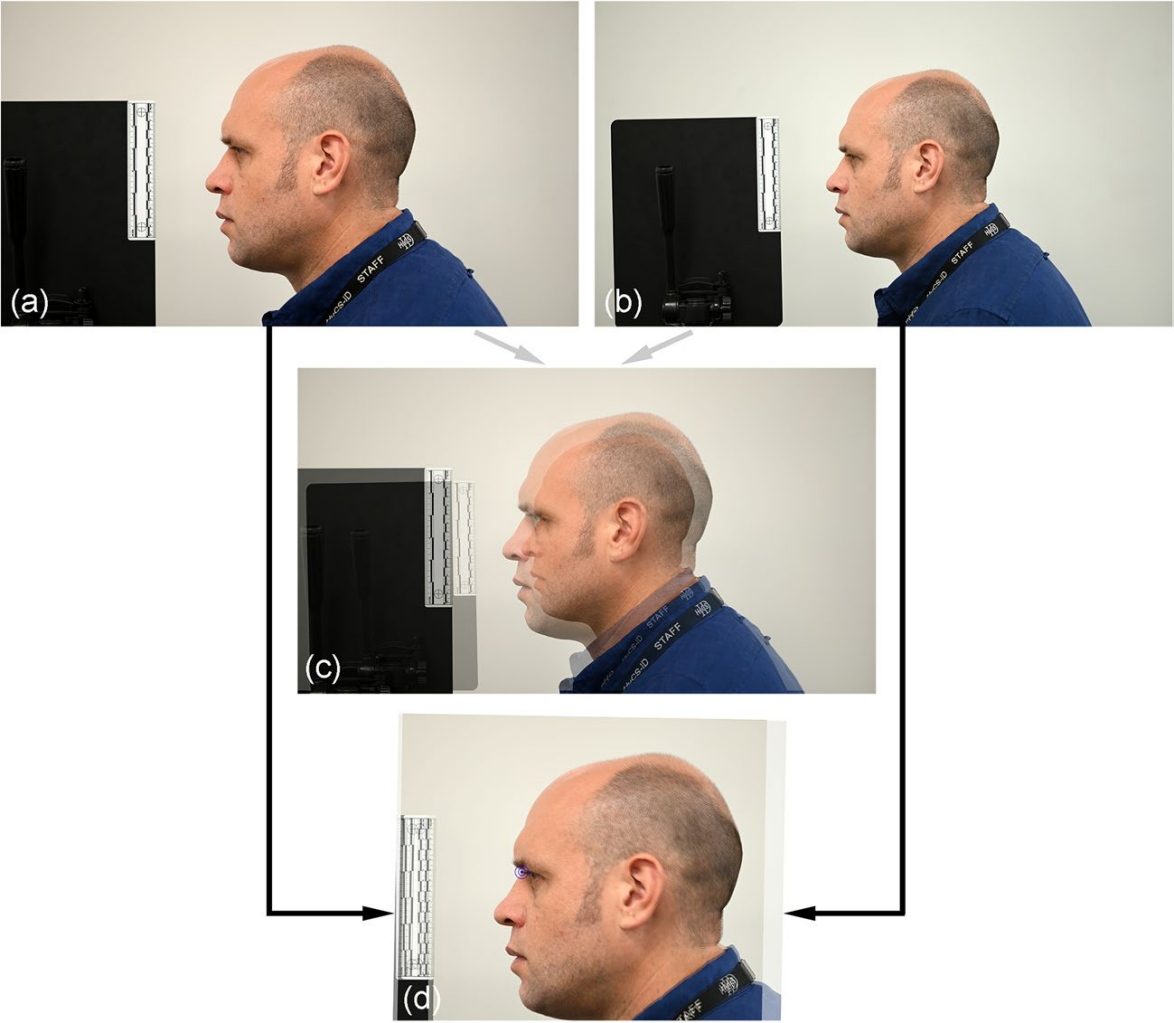
Example head scale differences at the ± 1% perspective difference limit, induced by difference focus distance. **a** Raw face photograph at focus distance of 2.0 m acquired with a Nikon^®^ D780 camera body fitted with a Nikon^®^ AF-S 105 mm f/2.8 prime lens. **b** Raw face photograph at focus distance of 2.4 m acquired with a Nikon^®^ D780 camera body fitted with a Nikon^®^ AF-S 105 mm f/2.8 prime lens. **c** Superimposition of images **a** and **b** without any fixed-aspect-ratio scaling. **d** Superimposition following fixed-aspect-ratio scaling (per all superimpositions conducted in this review) such that the two metric scales hold the same length/size, and in this case, with image registration at sellion per Fig. 3 (middle of bottom row)

A supplementary finding of the superimpositions conducted here was that fixed-aspect-ratio scaling of the second photograph in the superimpositions was required, including for the − 1% perspective difference condition (Fig. 5). Subsequently, we used a metric scale in all photographic images taken here to ensure exact fixed-aspect-ratio size adjustment (see, e.g., Fig. 5). However, this item indicates that even with a ≤ 1% difference in perspective mismatch, a focus distance difference under 4 m is large enough to produce large size differences between images that require correction. This is a complicating factor for craniofacial superimposition casework since, at the time of this writing, there is no data driven procedure to objectively determine what fixed-aspect-ratio scaling is necessary. This would be especially helpful in the craniofacial superimposition context because the skull and face do not represent homologous structures, so trying to fixed-aspect-ratio scale them to the correct size, by eye, invites error.

## Demonstration of perspective mismatch impact on superimpositions for a ground-truth skull/face pair

To illustrate how perspective distortion affects a true face/skull pair, we extracted the skull and face from a single CT scan image of a living subject (CS), which enables the comparison of the skull at its native position beneath the face, but at different focus distances to that used for face acquisition. This was accomplished in Blender [45] using the following procedure. First, the skull and face 3D models were rendered from the CT in OsiriX [44], then exported in standard tessellation language (STL). The 3D STL models were imported into Blender [45], where (a) the scale for each model was adjusted from meter units to millimeters; (b) the face and skull models were moved to a center position within the 3D virtual scene with the two endocanthions at an y-coordinate value of zero; and (c) the color tone of the face model was altered (to give a better contrast and visualization of the skull). Virtual cameras were serially placed at different known focus distances from the skull (i.e., on the y-coordinate axis), using the endocanthion as the zero point for the distance measurements. Back, key, and left-of-subject lights were added to the skull to improve its visual clarity and contrast (as the raw CT render is very uniform making the skull somewhat difficult to see). For partial profile and profile views of the head, the skull and face models were rotated 45° and 90°, respectively, and repositioned, such that the left endocanthion was again fixed at the zero value for the x-coordinate.

So that fixed-aspect-ratio scaling of the skull was not required (as the focus distance changed), the synthetic photographs were generated using lenses of multiple different focal lengths. The focal length was set by using a 2D square (orthogonal to the line-of-sight of the camera) in the image as a scale and selecting a focal length that maintained the size of square across all synthetically generated photographs at different focus distances. The 2D square was placed in the center of the virtual camera’s field-of-view, just like the skull, and on the coronal plane that intersected with hard tissue nasion. Note here that focal length of the lens does not impact the perspective distortion because perspective is determined by the camera vantage point alone and no other factor [20, 39, 42, 43]. When only the skull and face model were required to be viewed, the 2D square’s visibility was simply turned off, so that it disappeared.

For the 1:1 comparisons where the skull and face held the same perspective, we used a common focus distance of 4 m for both camera views of the skull and the face. From Blender’s [45] virtual cameras with set focus distances, 2D synthetic images were generated. A series of 378 additional synthetic images of the skull were generated each at a serially decreasing focus distances to 0.85 m, providing a range of synthetic perspective-mismatched skull photographs between 0 and 10% based on a mean facial height cord length of 179 mm. Focus distances of 3 m yield a perspective mismatch of 1% when compared to the face viewed at a 4 m focus distance. The synthetic skull photographs were superimposed with the synthetic face photographs at opacity so that the perspective mismatch could be visualized both in still and motion picture format (see supplementary online files S1 [frontal view], S2 [partial profile view], S3 [profile view], and S4 [all views]). As the face and skull meshes were derived from the same single CT scan, no subjective registration was required for the 4 m synthetic skull photograph (registration was exact to the ground truth). For each of the shorter focus distances, the skull was registered to the same xyz coordinate as hard tissue nasion on the 4 m skull photograph.

**Fig. 6:**
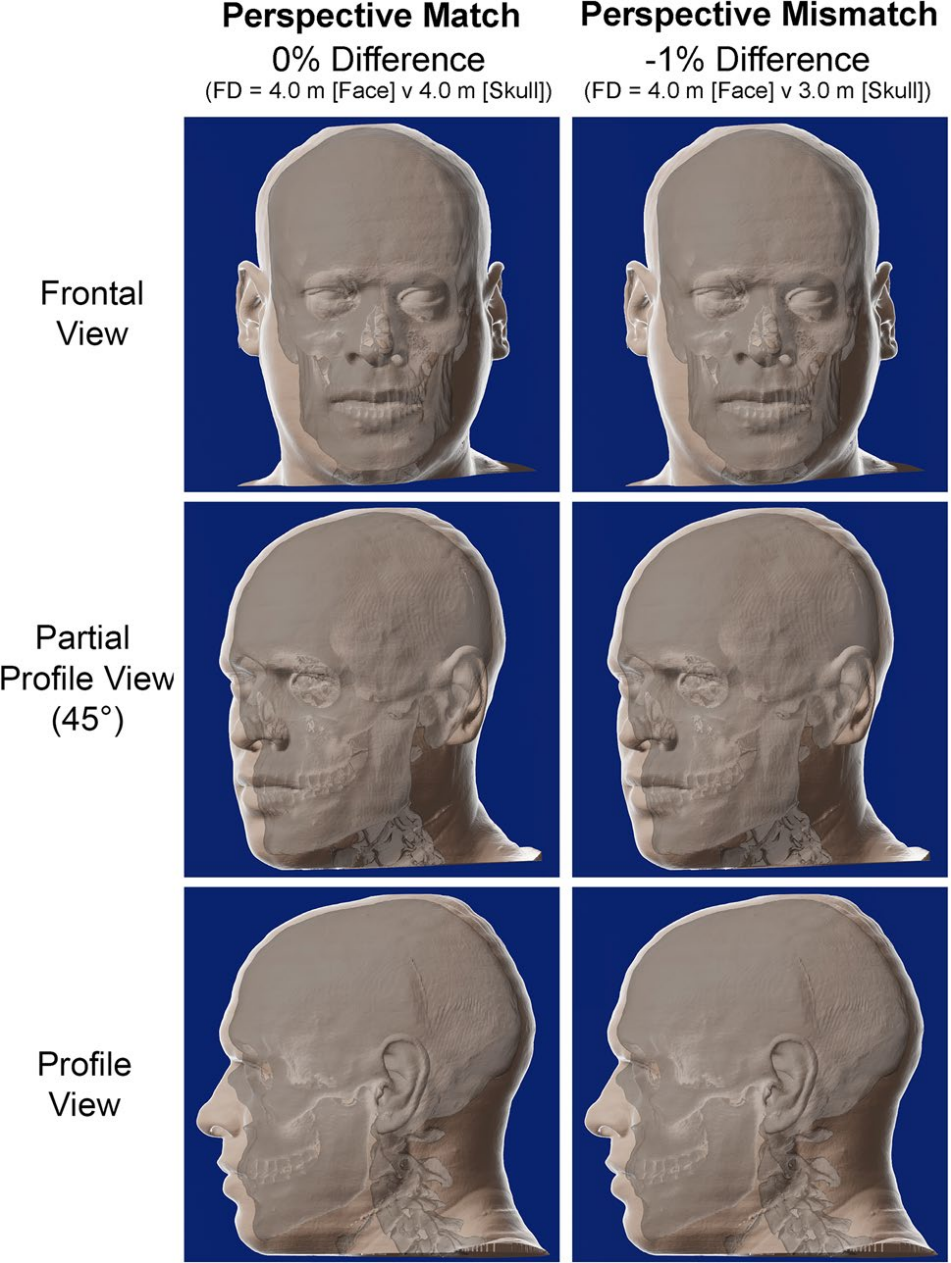
Superimpositions of synthetic photographs generated from a ground truth skull/face pair per the original CT scan of the living individual (CS). In the left column, the same focus distance (4 m) is used for the skull and the face, so there is no perspective mismatch. In the right column, a shorter focus distance has been used to view the segmented skull, generating a 1% difference in perspective between the synthetic face and skull images. In both superimpositions, the skull has been registered at the ground-truth nasion position evident from the native CT scan. Note that the changes induced by the 1% perspective mismatch condition are visually detectable in contrast to the 0% condition. For larger discrepancies in anatomy with larger perspective differences, see supplementary online files S1-4. A skull opacity of 35% has been used to create the superimpositions. FD = Image focus distance. Synthetic photographs generated in Blender® [45], from OsiriX [44] exported skull/face meshes and superimposed in Adobe^®^ Photoshop 2021 (San Jose, USA)

Figure 6 showcases, with still-frame images, the perspective mismatch at 1% relative to the 0% reference images. Here, it can be seen that there is almost no detectable visual difference between the 0 and 1% perspective mismatch images of the frontal, partial profile, or profile views. This supports the ± 1% perspective difference criterion and joint nasion/sellion registration as sufficient for craniofacial superimposition.

## Conclusions

A review of the previously posited ± 1% tolerance for perspective mismatch in craniofacial superimposition, using real-life photographs of the face and synthetically generated skull and face images from a CT scan, confirms the ± 1% tolerance level as sufficient for craniofacial superimposition. This ± 1% tolerance provides an allowance for a small amount of error to be present in the focus distance estimation, such that an exact value is not required for the superimposition to be feasibly undertaken. With some perspective mismatch tolerated, image registration becomes an important factor to minimize the perspective difference at the face (the principal region-of-interest). Greater attention should be awarded to focus distance estimation and image registration in craniofacial superimposition workflow to increase the scientific validity of the craniofacial superimposition procedure and reduce its subjectivity in the future. These steps should be undertaken prior to other attempts to align the skull to the face by rotation around the xyz axis. At focus distances yielding a ± 1% perspective mismatch, it is likely that some fixed-aspect-ratio scaling of the skull/face images will be required as part of the skull alignment procedure. Data driven methods to systematically determine the degree of fixed-aspect-ratio scaling required should be investigated in future investigations.

### Supplementary Information

Below is the link to the electronic supplementary material.

Supplementary file1 (MP4 34053 KB)

Supplementary file2 (MP4 34388 KB)

Supplementary file3 (MP4 32271 KB)

Supplementary file4 (MP4 18954 KB)

## Declarations

### Disclaimer

The views and opinions expressed herein are entirely those of the authors. They are not to be construed as official views of any institutions, editorial boards, or governing boards to which the authors may be affiliated.
